# EXPANSION: a webserver to explore the functional consequences of protein-coding alternative splice variants in cancer genomics

**DOI:** 10.1093/bioadv/vbad135

**Published:** 2023-09-26

**Authors:** Chakit Arora, Natalia De Oliveira Rosa, Marin Matic, Mariastella Cascone, Pasquale Miglionico, Francesco Raimondi

**Affiliations:** Laboratorio di Biologia Bio@SNS, Scuola Normale Superiore, Piazza dei Cavalieri 7, Pisa 56126, Italy; Laboratorio di Biologia Bio@SNS, Scuola Normale Superiore, Piazza dei Cavalieri 7, Pisa 56126, Italy; Laboratorio di Biologia Bio@SNS, Scuola Normale Superiore, Piazza dei Cavalieri 7, Pisa 56126, Italy; Laboratorio di Biologia Bio@SNS, Scuola Normale Superiore, Piazza dei Cavalieri 7, Pisa 56126, Italy; Laboratorio di Biologia Bio@SNS, Scuola Normale Superiore, Piazza dei Cavalieri 7, Pisa 56126, Italy; Laboratorio di Biologia Bio@SNS, Scuola Normale Superiore, Piazza dei Cavalieri 7, Pisa 56126, Italy

## Abstract

**Summary:**

EXPANSION (https://expansion.bioinfolab.sns.it/) is an integrated web-server to explore the functional consequences of protein-coding alternative splice variants. We combined information from Differentially Expressed (DE) protein-coding transcripts from cancer genomics, together with domain architecture, protein interaction network, and gene enrichment analysis to provide an easy-to-interpret view of the effects of protein-coding splice variants. We retrieved all the protein-coding Ensembl transcripts and mapped Interpro domains and post-translational modifications on canonical sequences to identify functionally relevant splicing events. We also retrieved isoform-specific protein–protein interactions and binding regions from IntAct to uncover isoform-specific functions via gene-set over-representation analysis. Through EXPANSION, users can analyze precalculated or user-inputted DE transcript datasets, to easily gain functional insights on any protein spliceform of interest.

**Availability and Implementation:**

EXPANSION is freely available at http://expansion.bioinfolab.sns.it/. The code of the scripts used for EXPASION is available at: https://github.com/raimondilab/expansion. Datasets associated to this resource are available at the following URL: https://doi.org/10.5281/zenodo.8229120. The web-server was developed using Apache2 (https://https.apache.org/) and Flask (v2.0.2) (http://flask.pocoo.org/) for the web frontend and for the internal pipeline to handle back-end processes. We additionally used the following Python and JavaScript libraries at both back- and front-ends: D3 (v4), jQuery (v3.2.1), DataTables (v2.3.2), biopython (v1.79), gprofiler-officia l(v1.0.0), Mysql-connector-python (v8.0.31). To construct the API, Fast API library (v0.95.1) was used.

## 1 Introduction

Alternative splicing is a widespread and complex process in eukaryotic cells that plays a critical role in regulating gene expression and generating protein diversity (Nilsen and Graveley 2010). The process is regulated by various factors, including cis-acting elements, trans-acting factors, and epigenetic modifications, which work together to determine which exons are included or excluded from mature mRNA (Black 2003). Dysregulation of alternative splicing has been implicated in the pathogenesis of various diseases, including cancer, as it can lead to aberrant protein isoforms that may have oncogenic properties or result in the loss of tumor suppressor activity (Kahles *et al.* 2018), for e.g. roles of alternative splicing of the CD44 and BCL2L1 genes in various types of cancer (Brown *et al.* 2011, Jiang *et al.* 2014). RNA sequencing (RNA-seq) technology has greatly enhanced our ability to study alternative splicing and its functional consequences (Merkin *et al.* 2012, Wang *et al.* 2015). Transcriptomics data can be used to identify differentially expressed (DE) transcripts, including alternative splicing isoforms, and investigate the functional implications of alternative splice (AS) variants, particularly in the context of cancer. Integration of transcriptomics data with protein functional annotations can help investigate the impact of alternative splicing.

Several methods and associated webservers have integrated the information of protein structures, domains, post-translational modifications (PTMs) and interaction networks to explore the consequences of genetic variants [e.g. Mechismo (Betts *et al.* 2015, Raimondi *et al.* 2019), Mechnetor (González-Sánchez *et al.* 2021), DysSysNet (Mosca *et al.* 2015)]. Similar efforts leveraged the information of protein domains and interaction network to predict the functional consequences of aberrant splicing events in cancer, suggesting a potential role as oncodrivers for some of them (Climente-González *et al.* 2017, Kahraman *et al.* 2020). A few webservers have also been recently proposed. DIGGER (Louadi *et al.* 2021b) integrates protein–protein interactions (PPIs), domain- and residue-level interactions information to interpret exon expression to a network level. CanIsoNet (Lay Karakulak *et al.* 2023) allows to analyze isoform switching events in multiple diseases, by integrating isoform expression data with interaction networks from STRING and ClinVar annotations. Similarly, NEASE detects protein domains affected by alternative splicing and then uses PPIs, domain-domain and domain-motif interactions to identify interaction partners likely affected (Louadi *et al.* 2021a). Additional online resources include ASCancer Atlas (Wu *et al.* 2023), a comprehensive knowledgebase of aberrant splicing in human cancers with highly curated cancer-associated splicing events, and their regulatory networks, which have been experimentally shown to promote tumorigenesis.

Here we present EXPANSION, a new webserver to explore the functional implications of AS variants, which integrates alternative transcript differential expression, domain architecture and PTMs annotations, isoform-specific protein interaction networks and gene enrichment analysis. The pipeline provides researchers with a comprehensive toolkit, both web- and API-based, to explore the functional effects of alternative splicing events in the context of cancer, as well as in any other condition of interest by allowing the upload and analysis of user-specified DE input dataset.

## 2 Methods

### 2.1 Retrieval of protein-coding transcripts and clustering

A list comprising 19232 protein-coding genes was acquired from HGNC. This list was used to execute queries on the EnsemblDB database, utilizing the ‘EnsDb.Hsapiens.v86’ package within the R environment to retrieve transcript related annotations (Rainer *et al.* 2019). CD-HIT (Li *et al.* 2001) was utilized to execute sequence clustering based on similarity. Using a threshold of 0.6, we successfully grouped closely related protein sequences that likely fulfill analogous functional roles. The clustering was crucial to avoid the alignment of sequences of substantially different proteins originated from the same genetic locus, e.g. Guanine nucleotide-binding protein G(s) subunit alpha, Neuroendocrine secretory protein 55 and ALEX, all coded in the *GNAS* locus. Since performing MSA on such sequences would lead to variation linked to different protein identity, and not to splicing, we first had to group sequences via clustering, so as to produce MSAs instrumental in detecting alternative splicing variation. We named each cluster based on gene symbol and the Uniprot Accession of the longest, canonical sequence in that cluster.

### 2.2 Multiple sequence alignments (MSAs) and detection of splicing events

For the sequences in each cluster, we generated multiple sequence alignments (MSAs) by using ClustalOmega (Sievers *et al.* 2011) with default parameters. The MSAs were exploited to detect either inserted, deleted or divergent regions in the alternative isoforms with respect to the canonical Uniprot sequence. To identify any splicing events that could affect structural and functional sites, we mapped Interpro domain definitions (Hornbeck *et al.* 2015, Blum *et al.* 2021) and PTMs obtained from PhosphoSitePlus (Hornbeck *et al.* 2015) onto Uniprot canonical sequences.

### 2.3 Protein isoform-specific interactions and over-representation analysis (ORA)

We retrieved protein isoform specific interactions from IntAct (version 1.38.0, R4.2) (del Toro *et al.* 2022), including ‘direct’, ‘physical’, and ‘association’ interactions. For each interaction, we retrieved the information of binding regions, wherever available, and mapped it to splicing-variable regions obtained from MSAs, to identify interaction sites affected by alternative splicing.

We integrated gene set over-representation analysis (ORA) to identify specific biological processes affected by alternative splicing. The g: profiler-official (v1.0.0) (Raudvere *et al.* 2019) python package was used to perform ORA of the functional categories significantly enriched in either the whole, or isoform-specific PPI network.

### 2.4 Integration with transcriptomics DE data

We integrated such protein-centric analysis modality with transcriptomics data, by using RSEM transcript abundances from the TCGA and GTEX databases (16 tissue-types, 19109 samples) which were processed through the TOIL pipeline (Vivian *et al.* 2017), and which we retrieved from the UCSC Xena Browser (Goldman *et al.* 2020). We used EBSeq, a Bayesian approach that provides more accurate estimates of differential expression compared to traditional methods (Leng *et al.* 2013), to identify DE transcript isoforms. Instances with a posterior probability of being differentially expressed (PPDE) greater than 0.95 were considered significant.

### 2.5 API

Through an application programming interface (API), we enable direct open access to data. The Python programming language and the Fast API library (https://fastapi.tiangolo.com/lo/, v0.95.1) were used to construct the API. The API documentation website (https://expansion.bioinfolab.sns.it/api/) offers information about how to use the API and the supported methods. The EXPANSION API is intended for multi-gene queries of the information integrated in the EXPANSION webserver and allows for retrieval of either individual, or combined, analyses generated for the queried transcript sets.

## 3 Web-server usage

**Figure 1.**
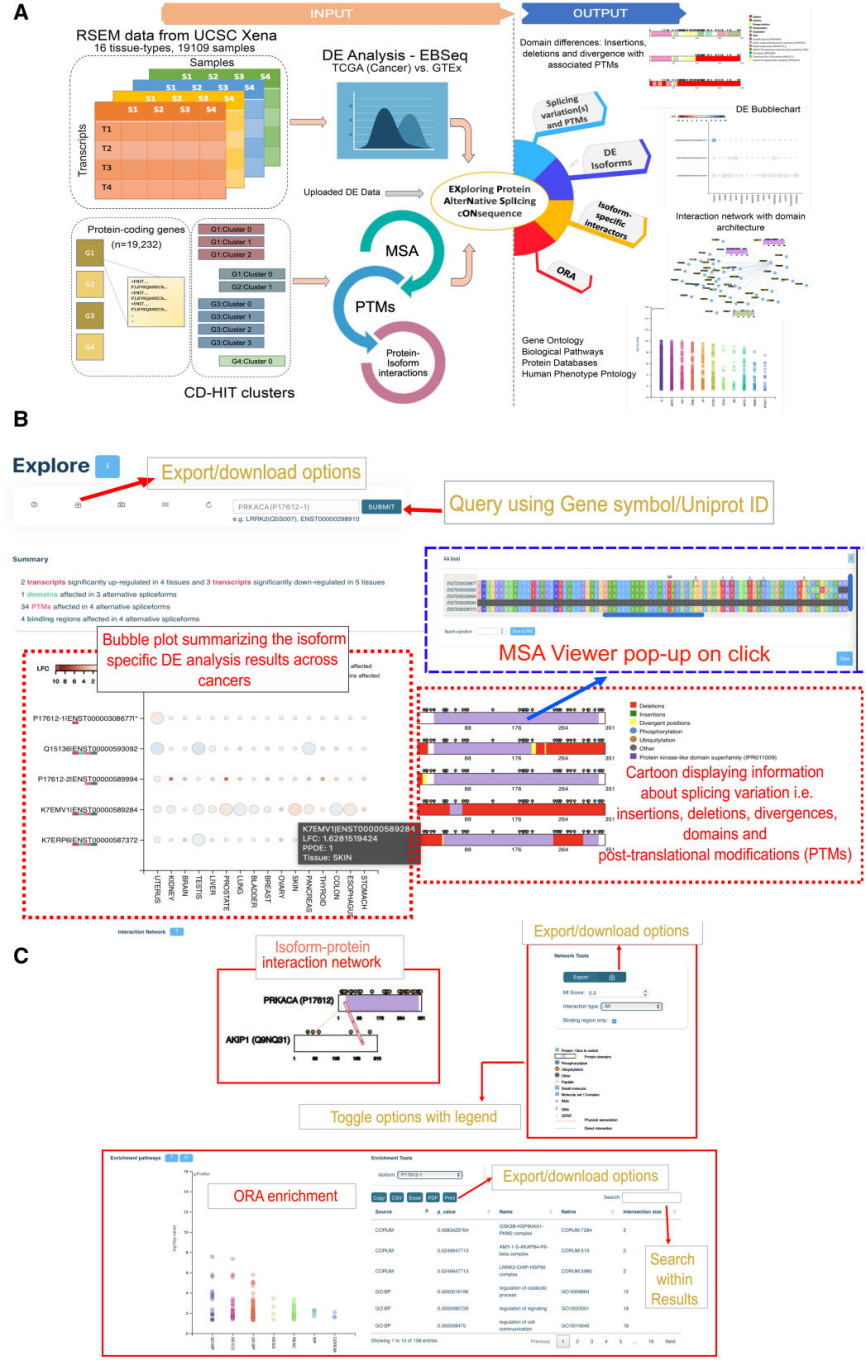
(A) Workflow and web-server functionality. The figure showcases the step-by-step process and various tools available to analyze protein AS-forms and their functional consequences, enabling users to query precalculated datasets, upload their own data, visualize transcript expression, explore splicing variations, examine protein interactions, and perform over-representation analysis of functional categories. (B) Snapshot of the first half of the main landing page when a user submits or uploads a query. Main features include (i) search bar for accessing isoform clusters. (ii) A color-coded summary of statistics for various assessments. (iii) Bubble plot visualizing differential expression of transcripts in each cancer tissue. (iv) Splicing variations and PTMs displayed in cartoon panels, (v) a click to open pop-up window to visualize MSA, and (vi) download options. (C) Snapshot of the second half of the main landing page when a user submits or uploads a query displaying (i) interactions mediated by protein isoforms shown based on IntAct data. Users can adjust interactors using toggles such as MI score and type of interaction. Region-specific interaction edges are highlighted if the splicing event affects the binding region and (ii) over-representation analysis (ORA) of functional categories for genes in the network. Comparison of biological processes mediated by isoform-specific interactors. Each of these is complemented with export options.

The web server provides a graphical framework to analyze protein AS-forms and their functional consequences in a gene-centric manner, as outlined in Fig. 1A. The ‘Help’ and ‘About’ pages contain detailed instructions, and users can query precalculated datasets (TCGA versus GTEX from Xena/TOIL) or upload their own differential expression (DE) transcript dataset. The format requirements for the uploaded DE transcript dataset are also provided in the ‘Help’ page. The search bar on the landing page allows users to access all available isoforms for a given gene symbol or ENSEMBL transcript id, grouped into clusters identified by the gene symbol and, in parenthesis, the uniprot accession of the canonical sequence representing that cluster (Fig. 1B).

After loading or querying the dataset, users can examine the differential expression of transcripts in each cancer tissue using the bubble plot (Fig. 1B). Each circle represents a transcript colored based on log-fold change and size proportional to DE significance (e.g. PPDE). The cartoon panel on the right displays splicing variation details, including insertions, deletions, divergent variations, and PTMs, using colored boxes and lollipops placed along the protein sequence architecture diagrams. The transcript structure can be explored interactively by double-clicking on the sequence diagram, revealing a pop-up window showcasing aligned transcript sequences from different isoforms, with a specific focus on the selected position. Furthermore, PTMs are highlighted using asterisks within the MSA viewer's pop-up window, offering an additional layer of insight to the visualization. Users can export both the bubble plot and the domain architecture diagram in SVG or PNG format.

The central panel displays interaction networks mediated by the protein isoforms based on interaction data from IntAct. Users can adjust the number of interactors using toggles for MI score, interaction type, and maximum number of isoform-specific interactors (Fig. 1C). Each node represents a protein, and its domain architecture can be visualized by clicking on the node to expand it. The node colors are representative of isoform specificity. Region-specific interaction edges are drawn if binding region information is provided, with corresponding edges highlighted in red if a splicing event affects a binding region.

Finally, users can visualize over-representation analysis (ORA) of functional categories for the genes in the network. User can specify whether to calculate ORA for either the entire, or isoform-specific networks, allowing comparison of distinct biological processes mediated by isoform-specific interactors (Fig. 1C). The results of the enrichment analysis are presented in a table that includes the genes linked to each significant term. The analysis excludes Uniprot accessions associated with alternative isoforms lacking distinct interactors.

### 3.1 *PRKACB* isoforms as a case study

PRKACB, a protein kinase A catalytic subunit, is characterized by alternative splicing isoforms, which are expressed in a tissue specific fashion and are associated to a number of disorders, including cancer (Ramms *et al.* 2021, Taylor *et al.* 2021).

**Figure 2.**
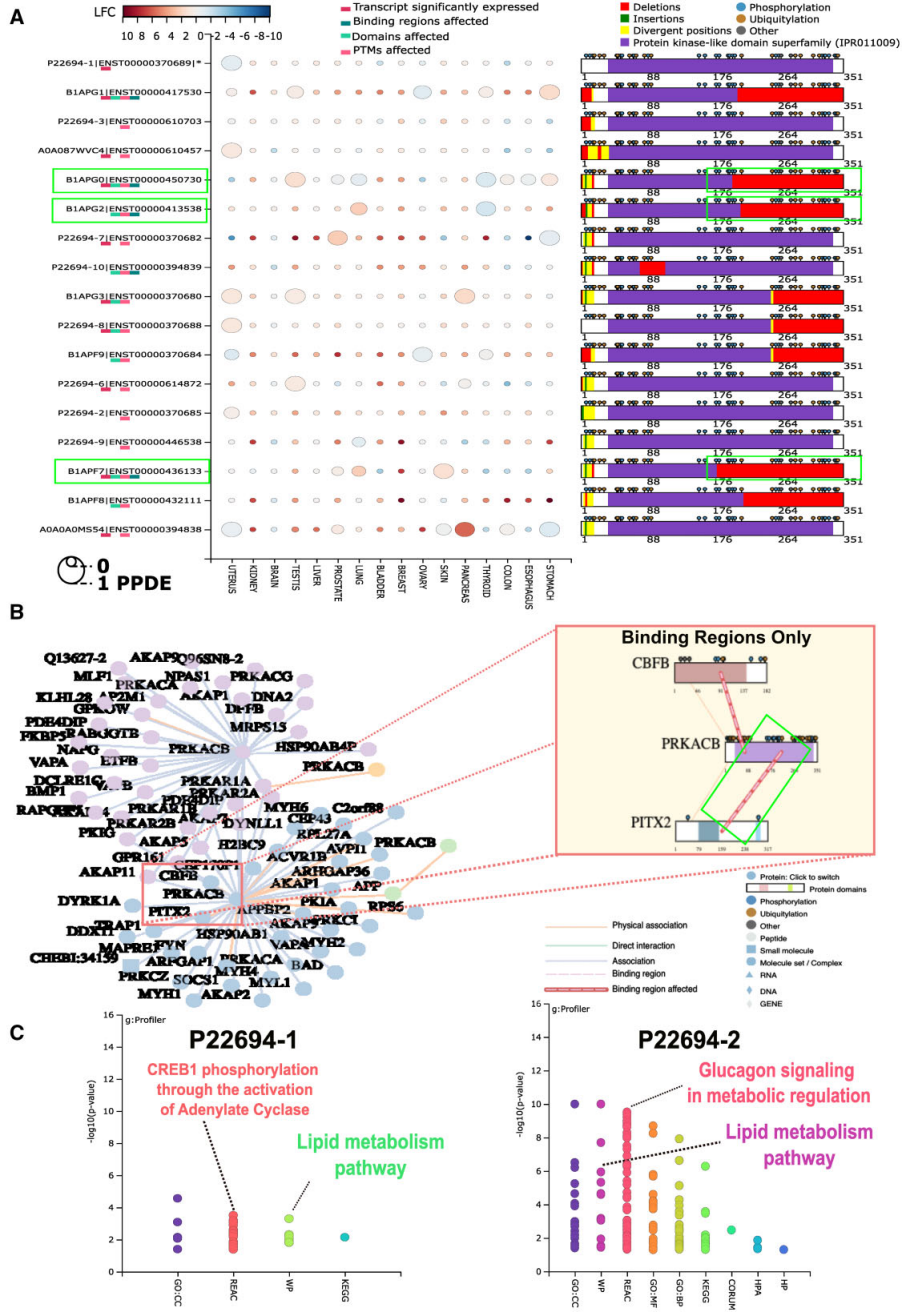
(A) Differential expression and protein domain changes in PRKACB splice variants. An analysis of differential expression for 16 PRKACB isoforms highlights significant changes in 9 isoforms within cancer samples, as denoted by larger bubbles in the bubble plot on the left panel. Five isoforms (e.g. ENST00000370680, ENST00000417530, ENST00000450730, ENST00000436133, and ENST00000370688) display C-terminus deletions affecting the ‘Protein kinase-like domain superfamily’, potentially influencing protein function and interactions, as shown in the domain architecture diagram on the right panel. Three of these isoforms (ENST00000417530, ENST00000450730, and ENST00000436133) exhibit modified binding regions with the interactor PITX2 and are shown in highlighted boxes. (B) Modified PRKACB Isoform Interactions with PITX2. The network of PRKACB isoform-specific interactions reveals alterations in interaction partners due to splicing variations. On the left, the full interaction network of PRKACB from Intact is shown using default settings. On the right the altered interactions due to binding regions are revealed when ‘binding region’ only is selected. Altered PRKACB-PITX2 interactions suggest potential downstream effects on signaling pathways and cellular processes governed by both proteins. (C) Overall Enrichment Analysis (ORA) of PRKACB Isoform-Specific Interactions. Enrichment analysis uncovers distinct terms for isoforms with available interaction information, such as P22694-1 and P22694-2, offering insights into potential biological roles linked to specific PRKACB isoforms.

We analyzed them via ‘EXPANSION’, including a set of sixteen reported PRKACB isoforms, nine of which show significant differential expression in cancer tissues. These isoforms, namely ‘ENST00000370680’, ‘ENST00000417530’, ‘ENST00000436133’, ‘ENST00000614872’, ‘ENST00000370688’, and ‘ENST00000610457’, are exclusively upregulated in different tissues; transcripts ‘ENST00000450730’ and ‘ENST00000370682’ are both up and down-regulated based on tissue types; while ‘ENST00000394838’ is exclusively down-regulated in Stomach cancer (Fig. 2A). Notably, 5 of these isoforms, i.e. ‘ENST00000370680’, ‘ENST00000417530’, ‘ENST00000450730’, ‘ENST00000436133’, and ‘ENST00000370688’, exhibit C-terminus deletions affecting the ‘Protein kinase-like domain superfamily’ (Fig. 2A). These deletions may lead to functional impairments, potentially disrupting protein activity and interactions.

The network of PRKACB-isoform specific interactions divulges which interactions (or interactors) are most likely to be affected in terms of splice-variants (Fig. 2B). Interestingly, among the nine different DE isoforms, three (‘ENST00000417530’, ‘ENST00000450730’, and ‘ENST00000436133’) display altered binding regions for the interactor ‘PITX2 (Q99697)’. PITX2 is a well-known transcription factor that plays a crucial role in embryonic development and tissue differentiation, particularly in the heart, eye, and pituitary gland (Chinchilla *et al*. 2011). Aberrant expression and functional alterations of PITX2 have been implicated in various types of cancer. In certain contexts, PITX2 acts as a tumor suppressor by regulating cell cycle progression, apoptosis, and DNA repair mechanisms. Conversely, in other instances, PITX2 can promote tumor growth and metastasis by enhancing cell survival, angiogenesis, and invasion (Fung *et al*. 2012). In addition, PITX2 has been associated with cancer stemness and drug resistance, further emphasizing its impact on tumor behavior (Zhang *et al*. 2013). The modified PRKACB-PITX2 interaction observed in these isoforms suggests a potential impact on downstream signaling pathways, gene expression, and cellular processes regulated by both proteins (Cox *et al*. 2002).

Furthermore, we discovered that the splicing-affected binding regions (210–218) within these three isoforms also exhibit an otherwise missing ubiquitination at position ‘214’ (Fig. 2A). This aberrant ubiquitination event is unique to these isoforms and may contribute to the altered dynamics of PPIs. The alteration of ubiquitination at this specific site could have implications for protein stability, degradation, or other regulatory mechanisms. Incidentally, these three isoforms lack interactor information in Intact, which results in no ORA enrichment results. However, for those isoforms for which this information is available for, e.g. P22694-1 and P22694-2, distinct enrichment terms are found which might hint toward specific biological roles (Fig. 2C). In particular, the PRKACB canonical isoform (P22694-1) is associated to cellular components such as ‘cAMP-dependent protein kinase complex’ (FDR = 2.7E^−5^) and pathways such as ‘CREB1 phosphorylation through the activation of Adenylate Cyclase’ (FDR = 3E^−4^), while the alternative isoform P22694-2 is associated to cellular compartments such as ‘Cyliary base’ (FDR = 1E^−10^) and processes such as ‘Lipid metabolism pathway’ (FDR = 1E^−10^).

## 4 Discussion

Overall, EXPANSION web server takes gene-centric queries, and provides a multi-modal annotation of alternative splicing isoforms to help predicting the functional impact of such variation. This analysis encompasses the identification of variants with respect to aligned sequences, domain architecture, PTMs, and interaction networks mediated by the protein isoform considered. Furthermore, over-representation analysis of functional categories is performed for each isoform-specific group to compare distinct biological processes mediated by isoform-specific interactors. These findings are then mapped onto the DE transcripts across cancers to obtain an integrated view of the functional consequences of DE spliceforms, whilst giving the possibility to process with the same pipeline additional DE transcript datasets. We also provide access to our webserver through an API interface, which allows for multi-gene queries.

EXPANSION currently includes only human isoforms and a possible area of improvement will be considering protein isoforms from additional model organisms. It would also be interesting to integrate experimental, isoform-specific interactions from IntAct with computationally predicted ones. This will be particularly useful for protein isoforms that are currently not reported in IntAct. Furthermore, incorporating methods into the webserver to predict the effect of splicing on PPIs would be intriguing [e.g. based on protein language models (Brandes *et al.* 2022) or graph neural networks].

While some of functionalities presented are in part included in other resources (see the Introduction for an overview), others, such as IntAct isoform specific networks are not. We therefore believe that our web-toolkit will offer researchers a comprehensive exploration of protein AS-forms and their potential functional consequences which is not fully available elsewhere, providing valuable insights for the study of these complex phenomena.
